# 平面と遊ぶ主体：アニメや漫画のキャラクター・イメージのトランスメディア的拡散

**DOI:** 10.12688/f1000research.129643.2

**Published:** 2023-10-13

**Authors:** 渡部宏樹 渡部

**Affiliations:** 1 Faculty of Humanities and Social Sciences, University of Tsukuba, Tsukuba, Ibaraki, 305-8577, Japan

**Keywords:** character, manga, anime, fan, film, kyara, audience participation, cosplay

## Abstract

Anime and manga characters are so ubiquitous in Japan that people see them anywhere, regardless of public or private. The two-dimensional flatness of line drawing allows the character images to straddle the boundaries of the worlds of works and media. The analysis of individual artworks needs to be revised to understand the transmedia spreadability of characters and our living conditions. This paper, thus, overviews the preceding discussions of anime and manga scholars on human-character relations and compares them with the theory of subject formation in film studies, whose realist tendency once emphasized the three-dimensional space and time. While psychoanalysis-influenced theories such as suture and male gaze model after one-point perspective and subordinate two-dimensionality, Thomas Lamarre formulates a de-unified perspective in relation to the multilayered image field of Japanese anime. In conjunction with the orientation of cultural and fan studies to emphasize audience agency, this article discusses the de-unified perspective on flat images which has the potential for understanding the complexity of subject formation through the interplay with two-dimensional images.

## 導入 : なぜキャラクターが重要なのか

日本で生活していると多くの場面で漫画やアニメのキャラクターに遭遇する。コンビニやスーパーマーケットに行けば食品、日用品、文房具、玩具などの商品がキャラクターの図像を付帯して販売されている( Steinberg, 2009, 130)。近年では安価な消耗品ばかりでなく、高級ブランドが漫画やアニメのキャラクターを利用することも珍しくない。営利企業に限らず、駅や行政施設などの公共空間でも注意喚起や啓発のためにキャラクターは多用される ( Nozawa, 2013)。地方の観光地に行くとゆるキャラと呼ばれる観光促進のキャラクターが見られ、また商業作品のキャラクターが起用されている例も見られる。観光資源に乏しい地方自治体が漫画やアニメのキャラクターのブロンズ像を建設し観光客を呼び寄せようという取り組みも珍しいものではなくなった。このようにキャラクターは、認知の向上とそれに伴う経済的利益を期待して公共的あるいは営利的な目的に利用されている。

キャラクターにかけられる期待は経済的なものに限らず、キャラクターが喚起する情動を用いた政治的動員効果も期待されている。例えば、安倍晋三首相は2016年にリオデジャネイロで行われた五輪の閉会式にスーパーマリオの姿で登場し、2020年に行われるはずであったオリンピックの宣伝を行った ( Rich, 2016)。国際的な観客に対するアピールとして彼自身より有名なマリオのキャラクターを利用し、同時に日本国内的にはポピュラー文化を利用した人気取りという側面もあっただろう。このようなキャラクターを利用する政治現象は日本に限らない。2016年の米国大統領選挙に際しドナルド・トランプの支持者たちは、「カエルのペペ」というキャラクターのイメージをトランプに重ね合わせたミームを作成し、このミームはトランプの勝利にも多少なりとも貢献したと言われている ( Bramesco, 2020)。

経済や政治において既に利用されていることをみれば、漫画やアニメの研究の一部としてではなく、キャラクターという現象それ自体の重要性は明らかであろう。「カエルのペペ」のミームがトランプ支持者らに利用され法的措置を通じて名誉を回復する過程を描いたドキュメンタリー映画『フィールズ・グッド・マン』（アーサー・ジョーンズ監督、2020年）は、結末で香港の雨傘革命で「カエルのペペ」が利用されていることをある種の希望として描き出していた。しかし、これはある特定の政治的立場からの価値判断であり、本質的にはキャラクター・イメージが力を持って人を動員するメカニズム、またひいては人がキャラクターのイメージを自己との関わりでどのように操作・利用するかのメカニズムの検討が必要である。

## 方法

この点を検討するために、本稿は、日本のアニメや漫画のキャラクター論をより広く主体とイメージの関係についての議論として捉え、関連するメディア研究、映画研究、ファン研究などの議論と比較検討する。まず、キャラクター論の画期となった伊藤剛『テヅカ・イズ・デッド』において、日本のキャラクター文化を可能にしているキャラクターが比較的簡単な線画であり容易に複製可能な平面的記号であることを確認し、伊東の議論の影響を受けた岩下朋世やマーク・スタインバーグらの議論を確認する。これらキャラクターの図像的特性についての議論と並行して、大塚英志と東浩紀がキャラクターを通した主体と世界の関係を議論していたことを取り上げ、キャラクター論をより一般的な主体とイメージの関係についての議論として再定式化する。その際に、三次元の時空間に優位を置いた英語圏の映画理論と比較することで、平面と空間という次元性が主体とイメージとの関係の中でどのように概念化されているかを整理する。これらの比較の中で、トーマス・ラマールがアニメの平面的な物質性に一点透視図法とは異なる主体と客体の関係を見出したことの重要性が浮かび上がってくる。脱一点透視図法的な主体とイメージとの関わりの具体的な実践の事例としてコスプレを捉えることができることを論じ、平面的なイメージとしてのキャラクターと鑑賞する主体との間に発生する複雑な関係性を明らかにする。

## 平面的キャラクターのトランスメディア的拡散

キャラクターが平面的な記号の組み合わせとして成立していることは漫画やアニメの研究者に限らず一般に広く理解されている。漫画家の手塚治虫は自作の中で、キャラクターは目や口、輪郭といったパーツの組み合わせで記号的に構成されていることを明示し後続の多くの漫画家に影響を与えてきた（ 大塚, 2003, 64-71; 大塚, 2009, 60-70）。本節では、漫画やアニメのキャラクターが記号的な図像であることを論じた漫画やアニメの研究者の議論を概観し、日本のキャラクター文化を可能にしている平面性についてまとめる。

まず多くの漫画・アニメの研究者が依拠する伊藤剛の「キャラ」と「キャラクター」を区別する議論を確認しよう。伊藤は、後述する大塚英志のキャラクター記号論や東浩紀の萌え要素のデータベース的組み合わせとしてのキャラクター論を受けて、登場人物のあくまで線画としての側面である「キャラ」とストーリーの展開の中で現れる人格面である「キャラクター」を区別した。伊藤は「キャラ」を次のように定義している。

キャラ : 多くの場合、比較的に簡単な線画を基本とした図像で描かれ、固有名で名指されることによって（あるいは、それを期待させることによって）、「人格・のようなもの」としての存在感を感じさせるもの（ 伊藤, 2005, 126）

この「キャラ」の存在感を背景にして統合的に立ち上がる人格的一貫性を「キャラクター」と伊藤は名付けているのだが、伊藤によるこの「キャラ」の定義には曖昧な要素がある。この点を指摘して岩下朋世は伊藤の「キャラ」概念を「比較的に簡単な線画を基本とした図像」と「人格・のようなもの」とに分割し、前者を「キャラ図像」、後者を「キャラ人格」と呼称し議論を精緻化した（ 岩下, 2013, 87-154）。ここで重要なことは、伊藤のキャラにしても岩下のキャラ図像にしても、これらの概念によって漫画の中の個々のコマにおける個々の線画を、作品世界内に立ち現れる一貫した人格的存在から切り離して議論の俎上に載せたことにある。

キャラクターの個々の図像的側面と人格的一貫性を分離して議論することで、作品世界の外側でのキャラクター・イメージの流通という現象を議論の俎上に載せることができるようになる。前述の通り、キャラクターのイメージは我々の家庭・学校・職場・公的施設といった生活のさまざまな側面に浸透し営利的・政治的・公共的目的を実現するために利用されているという事実が、現代日本におけるキャラクター文化現象を重要なものにしている。だとすると、漫画やアニメといったメディウム内在的な固有の記号作用の分析とともに、キャラクターがマーケティングや宣伝・広告に利用されるときにこれらを鑑賞する主体との間でいかなる感情・情動的関係性が発生するのかを分析する必要がある。

この点は、日本のアニメのメディア・ミックスの歴史を辿る中でマーク・スタインバーグが議論している。スタインバーグは日本初の商業テレビアニメ『鉄腕アトム』のキャラクターたちが、マーブル・チョコレートをはじめとする商品におまけのシールとして添付され、それによって作品世界を飛び出し人々の生活に浸透していたことを論じた（ スタインバーグ, 2015; Steinberg, 2009, 121-126）。日本のテレビアニメはリミテッド・アニメであり、線画で描かれる単純なキャラの静的なイメージが同時に運動性を持つことができた。このため、アトムのシールに見られるような、キャラクターのトランスメディア環境への拡散が可能になった（ スタインバーグ, 2015, 113-138）。スタインバーグが考察の対象とするのはアニメであり、伊藤と岩下が対象とした漫画とは異なるメディアである。漫画研究とアニメ研究を完全に同じ枠組みで論じて良いわけではなく、例えば、アニメ研究における原形質性（ 小山, 2022, 22-45）の議論は漫画研究に適用できる可能性は低い。しかしキャラクターに議論を焦点化する限りにおいては漫画とアニメを共通の土台において議論することは可能であろう。

伊藤は「キャラが間テクスト的に環境中に遊離し、偏在すること」を「キャラの自律化」（ 伊藤, 2005, 76）と呼び、このようにキャラが自律しトランスメディア的に拡散することを指摘している。スタインバーグが描き出している状況も「キャラの自律的」運動によって可能になったキャラのトランスメディア的拡散の一種であると言えるだろう。

キャラの「間テクスト的に環境中に遊離し、偏在する」特性が表れている文化現象の典型例が 2.5次元舞台である。2.5次元舞台では、「キャラの自律化」によってメディアを跨いで拡散したキャラクターの図像的側面が具体的な俳優の身体を通して発動する。須川亜紀子は、漫画やアニメの中でのキャラクターをめぐる複層的な記号作用の構造が、演劇という具体的な身体性を有したメディアに重なることでさらに多層化した現象として 2.5次元舞台を精緻に描き出している（ 須川, 2021, 22-38, 68-82）。3次元空間に肉体をもつ俳優自身も生身の人間としての顔と俳優としての姿を持つ複層的な存在であるが、彼ら/彼女らがキャラクターを模したメイクと衣装を纏い「キャラ図像」を獲得し、さらには「キャラクター」としての人格を演じることで、2.5次元舞台には複雑な意味の層が現れる。須川はこのような身体性を「ヴァーチャル・コーポラリティー(virtual corporality)」と呼び、1) 素の俳優の身体、2) キャラクターをまとったキャスト、3) 現象的肉体、4) 記号的身体、5) キャラクターといった 5つの層が舞台上にあらわれることを指摘している（ 須川, 2021, 73-82）。これら記号作用の層が「前景化・後景化を繰り返し、常に緊張関係にある」（ 須川, 2021, 81）ことが 2.5次元舞台の楽しみとなる。漫画やアニメの鑑賞と異なり、2.5次元舞台上に平面的な線画が存在するわけではない。しかし、観客たちは彼ら/彼女らが知っているキャラ図像を投影することで舞台上にキャラクターを認知的に現出させているのであり、原理的にはアトムのシールを家具や冷蔵庫に貼り付ける子供の行為と同じキャラをトランスメディア的に拡散させる二次創作的側面を持っている。

ここまで見てきた通り、キャラクターが比較的簡単な線画として平面的に図像化されていることが、そのトランスメディア的拡散を容易にしている。これは比較的簡単な線画で書かれていることが、キャラのトランスメディア的拡散の絶対に必要な条件であるという意味ではない。なぜならば、怪獣、戦隊モノ、巨大ヒーローなどの実写のキャラクターや生身の俳優やアイドルが、菓子、玩具、その他のグッズに印刷され流通し人気を博すという事例は多数存在するからだ。したがって、「比較的簡単な線画」という表現には、社会の中で技術的複製と運搬がどの程度容易に行えるかに依存するという条件が隠れている。パソコンやスマホなどが個人所有されデジタルデータを簡単に複製・運搬できる現在においては、「比較的簡単な線画」でなくともミームとして流通する可能性は高まっている。だが、初音ミクが人気を博した際に巨大なツインテールのイメージに単純化されて認識されたことで、絵心があるわけではない一般の人々も初音ミクのイメージのトランスメディア的拡散に関与できた（ 足立, 2020, 213-214）ことを考えると、技術的複製と運搬が容易な環境下でも「比較的簡単な線画」であることはイメージのトランスメディア拡散に寄与する重要な要素だと考えられる。

## 平面的キャラクターの向こう側の世界

このように自律化した平面的なキャラのイメージが社会の中への拡散するとき、そのキャラクターがもともと置かれる作品世界は完全に捨象されているのだろうか? 伊藤が参照した大塚や東の議論においてはキャラクターの図像的側面だけでなく鑑賞者が図像を介してどのように作品世界あるいは作品として表現される現実世界と関わっているかが論じられている。大塚と東の議論を追い、人間主体――文脈に応じて鑑賞者・観客・読者・視聴者・ユーザーとなりうる――のキャラクターを通した世界との関わりがどのように理論化されてきたかを確認しよう。

1989年に出版された『物語消費論』において、大塚英志は商品それ自体の使用価値ではなくその背後にある物語との関係で消費される事態を「物語消費」と名づけ、その事例としてビックリマンチョコレートに封入されるシールを取り上げた。ビックリマンチョコにはキャラクターが描かれた小さなシールが封入されており、シールの裏側にはそのキャラクターにまつわる断片的なエピソードが描かれている。シールは合計で 772種類あり、ある程度の枚数を集めてエピソードを収集すると、全体として叙事詩的な「大きな物語」が浮かび上がってくるという仕掛けになっている。大塚によれば、当時の子供たちは菓子を捨てて、キャラクターと物語が描かれたシールを収集するという「物語消費」をし、単にキャラクターのイメージを貼り付けただけの仮面ライダーのスナック菓子とは異なる消費形態であった（ 大塚, 2001, 7-22）。

子供たちはシールの表面に描かれたキャラクターの図像自体にも魅力を感じていた可能性は否定できないが、大塚の議論はキャラクターを作品世界との接点として捉え、キャラクターの向こう側に「大きな物語」たる作品世界があることに力点をおいている。2003年の『キャラクター小説の作り方』の中では、キャラクターを読者と作品世界との接点として捉え、そのキャラクターを通してみる特定の観点から作品世界が立ち上がることを強調する（ 大塚, 2003, 9-29）。

お話の中に「世界観」を見出さない、というのは近代という時代の、しかも「文学」という特殊なジャンルでの決まり事であって、同時にこの「世界観」に創作の比重をおくことが「文学」に対する「キャラクター小説」のもう一つの特徴とも言えます。

あれ、キャラクター小説の本質はキャラクターにある、とこの講座では前からしつこく主張してきた、と思うかもしれません。しかし「世界観」とは繰り返しますが「世界」の「観」方です。作中のキャラクターが「世界」をどのように「観」て、受け止めるかということがキャラクター作りにおいて不可欠であり、同時に「世界観」作りにおいて最も見落とされている点でもあるのです。[…]「世界観」とは読者がキャラクターの目を通じて「観」る「世界」でなくてはなりません。（ 大塚, 2003, 221）

ここには大塚のキャラクター理解が凝縮している。大塚にとっては個々のキャラクターよりもキャラクターを通して浮かび上がる大きな物語あるいは作品世界全体が重要となる。手塚が記号的に描かれるキャラクターの身体を傷つき得る、死に得るものへと変えたことを強調しすることで、大塚は漫画をリアリズム的問題系へと引きつけ、戦後民主主義的価値観を倫理的審級として漫画批評に導入した（ 大塚, 2003, 259-282; 大塚, 2009, 117-144）。この大塚の議論からは、キャラクター自体は記号的であるとしてもその向こう側に出現する世界が我々の現実と乖離したものでないことを要請する立場が必然的に導き出されることになる。

一方、大塚の『物語消費論』を参照し 2001年に出版された『動物化するポストモダン』の中で、東はキャラクターを欲望の対象あるいは結節点として捉えている。「物語消費」に対して提起される「データベース消費」においては、手塚の記号論的な観点を踏襲し、キャラクターは「萌え要素」の集合体として捉えられる。例えば、特定の髪の色や髪型といった個別の記号的要素が、ある特定の性格のタイプなり行動のパターンなりと紐づけられ、キャラクターを鑑賞するものはその特定の記号に対して「萌える」という感情的・情動的反応を行う。このような鑑賞あるいは消費の形態に必要なのは、キャラを構成する記号とその意味の組み合わせを列挙したデータベースであり、作品世界といった大きな物語ではない（ 東, 2001, 42-47, 50-58）。

しかし、東がキャラクターを消費する主体が世界とどのように関わるかを検討していないというわけではない。『ゲーム的リアリズムの誕生』では、キャラクターを複眼的な主体を立ち上げる契機として捉えている。東はノベルゲームと呼ばれる物語を文字情報として読むことを中心としたゲーム作品の中には、作品世界内で物語が何度も繰り返される特徴があることを指摘している。「スーパーマリオ」のような何度も挑戦できるゲーム的設定を有することで、これらノベルゲーム作品の中で、主人公は同じ物語を少しずつ違う立場や視点から何度も生き直す。東は、このようなキャラクターが何度も死を繰り返し生き直すことで到達する独特の現実感覚を「ゲーム的リアリズム」と呼び、写生文学的リアリズムとは別の現実に対する認識のあり方を強調した。ゲーム的リアリズムを体現した文学作品の中では「キャラクターがメタ物語的な結節点として与えられているがゆえに、あらゆる物語に対して別の物語への想像力が半ば自動的に開かれてしまう」（ 東, 2007, 125）のである。

大塚と東はそれぞれ異なる立場から、鑑賞者がキャラクターを通してどのように作品世界あるいはそこに表現される現実と向き合うかを議論した。大塚はリアリズムと戦後民主主義的価値観を倫理的審級として強調しキャラクターの向こう側に現実があるべきことを重視し、東はキャラクターの複製的な性質を媒介することによって単一の視点の位置に還元できない複眼的な世界との関係のあり方を模索している。本稿はこれらの立場の優劣を議論するものではない。なぜならば、大塚と東はともに作品を分析の単位として前提とした上で鑑賞者とその作品世界との関わりを中心に論じており、個別作品の批評に基づいた社会批評を行ってはいるが、キャラクターそれ自体は必ずしも関心の中心ではないからだ。一方、本稿が注目するのは作品世界の境界線を超えて我々の日常生活の中にキャラクターが氾濫しているという事実である。

足立加勇のキャラクターという現象が「人間の表象」であると同時に「ファンのコンテクスト」でもあるという二面性への指摘が、この対立を理解する助けになる。足立は、漫画の実作の中にキャラクターをあくまで人間の表象として理解しようとする自然主義的傾向がかつてあったという伊藤の議論をひいて、この自然主義は、「キャラ」が図像的現象であるという事実だけでなく「キャラ」というものが社会集団に共有されているという事実を隠蔽していることを指摘した（ 足立, 2020, 217）。つまり、大塚が論じているようなキャラクターをあくまで個人が世界に向き合うときの一つの観察の視点として捉える立場は、原理的に「比較的簡単な線画」であるキャラが多くの人に描かれ共有されるという事実を見落としてしまうということだ。ここで、足立は伊藤の議論を批判的に継承し、キャラクターを鑑賞する主体たちが共有されたイメージを通して社会的振る舞いの方に議論の力点を移している。

この観点は、キャラクター文化の理解に資するとともに、そのマーケティングや宣伝の道具としての有用性をも明らかにしている。キャラクターの図像的側面が物語内的一貫性から切断できることが、「キャラが間テクスト的に環境中に遊離し、偏在すること」を可能にしており、だからこそマーケティングや宣伝に利用することができる。2.5次元舞台においては舞台上のキャラクターを演じる俳優はもとの作品世界全体を呼び出すことを企図しており、ここでは大塚が描いたようにキャラクターを通して「世界」が立ち現れていると言える。一方、マーブルチョコレートに添付された鉄腕アトムのシールは『鉄腕アトム』の作品世界全体やその中でのアトムの喜怒哀楽の全てを呼び起こすことを目的としてはいない。2.5次元舞台と比べた場合、ここでは東の「萌え要素」の喚起力と同種のメカニズムが機能する余地が大きい。仮に消費者が物語の作品世界全体を味わうことだけを目的とするならば、その消費者はマーブルチョコレートには目もくれず漫画の単行本を所有することだけに喜びを見出すだろう。「萌え」ではないとしても、アトムの固有の図像的魅力が消費者を惹きつけているのだ。

このように考えると、伊藤のあいまいさを残したキャラの定義が深い洞察を伴っているものに見える。「多くの場合、比較的に簡単な線画を基本とした図像で描かれ、固有名で名指されることによって（あるいは、それを期待させることによって）、「人格・のようなもの」としての存在感を感じさせるもの」（ 伊藤, 2005, 126）という記述は、キャラの図像とそれが喚起するなんらかの人格や感情的・情動的状態との不可分性を示唆している。アトムのイメージが商業的に流用されるとき、それを鑑賞する主体になんらかの「「人格・のようなもの」としての存在感を感じさせる」からこそ、マーケティングや宣伝にキャラクターが多用される。

平面的なキャラクターのイメージが常になんらかの感情・情動を喚起することは、アニメの聖地巡礼、既存の漫画やアニメキャラクターを使った地域振興、日本全国で見られるゆるキャラの採用といった現象を考える際に示唆的であろう。漫画やアニメの舞台となった土地を訪問する観光の形態は聖地巡礼と呼ばれ、現在では地域振興のための手段として注目されている。しかし、漫画やアニメ作品の中で現実に存在する場所が取り上げられるというインデックス性がこの聖地巡礼を引き起こしている点で、キャラクターのメカニズムとは齟齬が生じる。だからこそ、日本各地で設立されている漫画やアニメのキャラクターの銅像は、その銅像が作者の出生地であるといった現実とのか細いつながりに頼る形で、漫画やアニメのキャラ図像が喚起する情動を観光客誘致の手段として使っている。ゆるキャラの場合はキャラクターの造形の中にその土地を示す記号を導入することで、キャラを土地に繋ぎ止めている。いずれの場合にしても、それらを何らかの形で具体的な土地とのインデックス的なつながりを作りながら、キャラが必然的に喚起する感情・情動が利用されている。

だとすると、キャラクター文化自体は原理的には地域に対する関心を引き起こさない。漫画やアニメのキャラクターの像を見に訪れる観光客は、ブロンズ像というキャラの図像によって喚起されるもの――大塚のシナリオであれば作品世界全体を、東のシナリオであれば記号的に喚起される感情・情動を――消費しているのであり、実際の観光地が作品の中に出現する場合を除いて観客は地域振興を仕掛ける側が売り込んでいる土地を見ているわけではない。ふるさと納税制度のようにさまざまな商品が並んでいて欲望を喚起するが、納税者は返礼品を提供する「ふるさと」に興味を持っているわけではないことと同じである。

本稿はコンテンツ・ツーリズムや地域振興の具体的な方法を検討することを目的としてはいないが、仮にキャラクター文化をこれらに利用しようと考えるのであれば、主体がキャラクターとの間に持っている感情的・情動的関係を、主体と振興しようとする土地との関係に変換するメカニズムを考える必要がある。だとすると、問題はやはり平面と主体の関係なのである。東の「萌え」はこのような主体と平面の関係を記述するひとつの用語である。本稿を執筆している 2022年時点では「萌え」はさほど使用されないが、「エモい」、「推し」、「尊い」といったイメージとの感情的・情動的関係性を定義する言葉は多数出現している。これらの用語はイメージが主体に対して喚起する感情に注目した概念であることからわかるように、キャラクター文化においては対象と主体とがどのような関係を結ぶかが問題となる。

## 英語圏の映画理論における3次元空間の優位

平面的対象とそれを鑑賞する観客主体の関係は、英語圏の映画研究においては観客とスクリーンの関係として議論されている。しかし、英語圏の映画研究においては、日本語でのキャラクターの議論とは異なり、平面性や無時間性には劣位が与えられている。日本のキャラクター論との比較のために、まずはこの点を確認しよう。

映画は 19世紀末に技術が完成し、20世紀初頭に見せ物として利用され、ハリウッド映画産業の国際的な覇権の拡大とともにアメリカ合衆国が生み出した独自の芸術形式と見做されるようになった。他のより古くから存在する芸術形式に対して映画を芸術として確立するために、映画というメディウムに固有の芸術的特性が何かが検討された。その有力な答えの一つはカメラが現実をそのまま映しとることである。カメラという機械の目が人間の意図とは関係なく、世界をありのままに空間的な広がりと時間的持続を伴って記録できることが称揚され、これが映画学におけるリアリズムの基礎となっている ( Stam, 2000, 72-83; 角井, 2021, 76-77)。もちろんこのように映画の特性を規定することは20世紀のある時点までの実践に過ぎないが、この映画的リアリズムという規範からは三次元的間が重視され、漫画やアニメのキャラクターは平面的で無時間的なものとして一段低く見積もられる。

映画研究が精神分析を取り入れる過程を見ると、英語圏の映画学における三次元空間への偏重は理解されよう。映画理論はジャック・ラカンの精神分析理論を導入し、観客主体が平面的なスクリーンを通して作品世界に没入するプロセスを理論化してきた。この主体形成のメカニズムは精神分析の用語を借用して「縫合」理論と呼ばれる。その理路は次のようになる。あるショットに対して想像的に同一化した観客は、このショットを撮っている全能の存在――「不在の他者」――が全体をコントロールしているのではないかと不安を抱く。だが、このショットに続く切り返しショットがそのショットを撮る位置に誰もいないことを露わにする。このショットと切り返しショットの連続によって、観客は映画に「縫合」され、主体となるという理屈である ( Oudart, 1989, 45-57; Dayan, 1974, 22-31)。精神分析における諸々の概念は関係論的ではあっても物質的に空間的ではない。この関係的概念を具体的な現実的な空間を重視する映画的リアリズムにひきつけた点が映画理論における「縫合」理論の要点となっている。つまり去勢不安が具体的な三次元空間状における「不在の他者」の占める位置へと翻訳され、ショットの連続によって「不在の他者」が存在しないことを示すことで、この不安を解消している。

縫合理論の影響を受けたフェミニズム映画理論においては、三次元空間の優位に加えて、二次元的な平面性の劣位が明確に表れている。例えば「物語映画と視覚的快楽」の中でローラ・マルヴィが定式化した「男性のまなざし」を見てみよう。「男性のまなざし」とはハリウッド映画において、男性主体が女性を性的対象としてまなざす構造を持っており、観客たちは映画の観客となることで異性愛体制を内面化するという議論である。観客がスクリーン平面上に映し出されたイメージを通してそのような規範を受け入れる過程は次のように説明される。ハリウッド映画の中での自由に動き回ることができる男性主人公は精神分析で言うところの「想像界的」な快楽を体現している。観客はこの男性主人公に同一化することで、物語の作品世界内に没入することができる。作品世界内での男性主人公の欲望の眼差しとそれと重ね合わされるカメラの眼差しを通して、観客はこの異性愛秩序を受け入れる( マルヴィ, 1998, 129-135)。この論理の中で女性の身体は男性の性的欲望の眼差しの対象としての固定した平面的イメージとして描きだされる。

お馴染みの女性の足のクロースアップ（例えばディートリッヒの）、もしくは顔のクロースアップ（ガルボの）もまた別の 性エロテ 愛ィシズムの様式を物語の中に統合する。クロースアップにより断片化された身体の部分は、物語が必要とするルネッサンス的な遠近法の空間と 画スク 面リーン上の奥行きの錯覚を無効にしてしまう。身体の断片は画面を平面的なものに変え、物語的な現実らしさよりはむしろ切り抜き絵か 肖イ 像コン的な肌合いをもたらしてしまうのである。( マルヴィ, 1998, 132)

ここに三次元の空間の優越と二次元の平面の劣位を読み取ることは容易だろう。個別の映画作品は物語として「ルネッサンス的な遠近法の空間と 画スク 面リーン上の奥行き」を要請し三次元の空間を有している。しかし、その物語の中で、クローズアップにより女性の身体は断片化され、平面的な「現実らしさよりはむしろ切り抜き絵か 肖イ 像コン的な肌合い」のものにされてしまう。この静的なイメージとしての女性身体が観客のまなざしをとらえるものとなり、「その途端、 呪フェ 物ティ 崇サイ 拝ゼーシ 化ョンが起こり、去勢不安を隠蔽しながら視線を凍結し観客を固着し、観客が自分の目前にある画像から距離をとるのを全面的に防いでしまう」( マルヴィ, 1998, 139) のだ。ここでマルヴィは、男性主体が女性のイメージに囚われた状態を「距離をとるのを全面的に防いでしまう」と空間的比喩で表現していることも興味深いが、「凍結」や「固着」といった運動性を欠いた静止した状態として描写していることも重要である。映画が時間芸術でありかつ三次元の空間性を重視するというリアリズム的前提のもとでは、運動を捉えることに対する肯定的評価が必然的に導かれる。その反作用として、マルヴィは運動性を欠いた非時間的なイメージを劣位に置く修辞を駆使している。

マルヴィの男性のまなざしの議論は広範な影響を与え、多くのフェミニズム映画理論家が彼女の議論の瑕疵を指摘した。例えば、ジョアン・コプチェクが指摘しているように、縫合理論は主に「私の機能を作るものとしての鏡像段階」の議論を参照しているが、後にラカンはその議論を大幅に修正している ( コプチェク, 1998, 29-58)。しかし本稿が注目したいのは、映画理論における縫合の議論がリアリズム的な前提によって精神分析を具体的な三次元空間の問題に議論を縮減し、結果として出現した二次元的な平面性を低く評価する論理がキャラクターの理解にどのように貢献するかである。

マルヴィの「視線を凍結し観客を固着し、観客が自分の目前にある画像から距離をとるのを全面的に防いでしまう」という記述は、ここまで述べてきた日本のキャラクター論における主体とキャラクターのイメージとの関係を描き出しているようにも読める。ある欲望のあり方へと固着させてしまうと言う点では、ハリウッド映画の中の断片化された女性身体のイメージと、東が「萌え要素」の集合体として描き出す日本のキャラクターは類似したものである。こういった喚起力があるからこそ、スタインバーグがキャラを切り出した理路のようにアニメのシールが魅力を発散するし、コンビニやスーパーマーケットでキャラクターが添えられた商品は後をたたない。しかし、ハリウッド映画の中で三次元の空間性と対比する中で平面のイメージを論じるマルヴィの理論を、そのまま日本の漫画やアニメのキャラクターに適用することは妥当だろうか?

## 分配的領域を見る脱一点透視図法的主体

トーマス・ラマールの『アニメ・マシーン』(2009 年) がこの問いに関連する重要な議論を行っている。同書はアニメを制作する際のマルチプレーン装置の構造に注目し、ハリウッド映画的な奥行き方向の運動性を持った一点透視図法的な視覚の体制である「シネマティズム」に対して、日本のアニメの特徴である奥行き方向の運動性を欠いた複数の平面間の関係性の美学としての「アニメティズム」を対比させたものである。したがって、キャラクター論がラマールの主要な関心というわけではない。しかし、三部構成の本書の第三部において、CLAMP原作の『ちょびっツ』を題材にアニメにおける「縫合」の問題を取り扱っている。

ラマールは映画の編集慣例に則って作られるアニメにおいては、縫合はある程度まで発生するが、最終的に縫合は破綻することを議論している ( ラマール, 2013, 349-354)。これはラマールが平面同士の関係性の美学を強調していることから必然的に導かれる結論である。縫合理論は、一点透視図法を実現する装置であるカメラという物理的機構から導き出されるリアリズム的規範を経由させて、精神分析という非物質的メカニズムを空間化した理論であり、ラマールが論じるアニメーションの技術的物質的特性はこれと異なる。複雑な立体的構造物を平面的に開いた展開図を見る時に、それを見るものは平面の各部分ごとの構造や仕組みを合成することで全体の出来事を理解するが、これがアニメの平面的美学で起こっている意味作用だ。ラマールはこのようなアニメの認知的メカニズムが発生する場を「分配的領域」( ラマール, 2013, 148-149, 179-182, 328-331) と呼ぶ。分配的領域において、一点透視図法が想定する単一の点としてではない主体の認知が立ち上がるというのが、アニメにおける観客とイメージとの関係である。もちろんアニメは映画の慣習や文法に則って制作されるため一定程度縫合的なメカニズムは発生するが、物質的な三次元空間の実在に依拠してある特定の点としてのまなざしの主体を立ち上げるメカニズムである縫合が、アニメにおけるイメージと観客主体の関係を規定しているわけではない。

とはいえ、ラマールはアニメの中でイメージが主体を形成するメカニズムが存在しないと言っているわけではない。視覚的モデルの代わりに力学におけるアトラクターとコーペレーターという議論を導入してこれを説明している ( ラマール, 2013, 329)。ラマールによると、アトラクターとは「ある力学的システムがそれに向かって発展していく集合」であり、それは点でも曲線でも多様体でもフラクタル構造でもありうる。例えば振動する振り子は摩擦を受けていれば十分な時間が経過したのちにある一点で静止する。この静止する一点が点アトラクターである。コーペレーターとは、「アトラクターに向かう発展に関与する機能」である。その上で、ラマールは次のように両者の関係を論じている。

多平面的なアニメ・イメージの平板化と結びついた分配的な領域という文脈では、萌え要素がアトラクターとして機能する。アトラクターは、諸要素が脱階層化されて分配される領土上で顕著となる萌え要素である。とすれば、相互作用の担い手であるオタクはコーペレーターである。情動的な環状回路や循環経路がコーペレーターとアトラクター、すなわち人を惹きつける萌え要素とオタクとをつなぐ。コーペレーター（あるいはインターアクター）は、その領域内にある、単なるもう一つの要素という以上のものであり、一つの視る位置以下のものである。それは、安定した視る位置や主体ではない。コーペレーターは、アトラクターとの関係で諸要素を統合し差異化する機能である。オタク・コーペレーターの小さな世界や小さな物語は、領域内に分配され密封された諸要素の相互作用を通じて創発する複雑なパターンなのだ。( ラマール, 2013, 329)

ここでラマールはおそらく厳密に力学的な意味でアトラクターとコーペレーターという単語を用いてはいない。アニメの分配的領域において「萌え要素がアトラクターとして機能」し「相互作用の担い手であるオタクはコーペレーターである」と言っていることから、画面上に配置された記号的萌え要素とオタクの鑑賞者のような、イメージと主体の関係の表現であると考えてよいだろう。ここで、注意すべきはアトラクターと相互作用を行うコーペレーターは分配的「領域内にある、単なるもう一つの要素という以上のものであり、一つの視る位置以下のものである」ことだ。このように言うことで縫合理論や男性のまなざし論が想定しているような「安定した視る位置や主体」とは異なることを強調している。ラマールは一点透視図法的な主体を立ち上げることを避けつつ、しかし同時に人間とイメージの間の関係性を定式化しようとしている。

その意味で、ラマールの議論はここまでの主体とイメージをめぐる議論の系譜に並ぶものである。ラマールは東の「データベース消費」の議論を踏襲しており、オタク・コーペレーターは記号的萌え要素によって刺激され発火するオタク主体の中の個々の感情的・情動的反応と理解することは可能であろう。だとすると、スタインバーグが議論したアトムのシールをアトラクター、それらを収集し家具や冷蔵庫に貼り付ける子供をコーペレーターと考えることもできる。このとき、アトムのシールを家庭内に貼り付ける子供はシールと室内を統一的にまなざす一点透視図法的視点ではなく、アトムのシールという「アトラクターとの関係で諸要素を統合し差異化する機能」を有しただけの存在である。同時に、ラマールの議論はマルヴィの議論とも意外なほどに近接している。クローズアップによって断片化された女性の身体の平面的イメージというアトラクターに対して、欲望するコーペレーターは異性愛男性の規範を内面化した観客の反応と理解することもできる。マルヴィが前提としていた三次元の優越のもとでは、クローズアップによって断片化された女性身体のイメージは「観客が自分の目前にある画像から距離をとるのを全面的に防いでしま」い、「物語が必要とするルネッサンス的な遠近法の空間と 画面スクリーン上の奥行きの錯覚を無効にしてしまう」という危機を招くものであるが、ラマールの「分配的領域」には劣位の二次元性という発想は存在しない。

以上のことから、アニメにおいて縫合――すなわち作品世界への没入――が十分に機能しないという議論は、別の言い方をすると、キャラクターの目による世界観を通して作品世界全体を鑑賞する大塚的なモデルの機能不全を示している。このことは日本におけるキャラクターの受容の現実をよく反映していると言える。例えば、「倍速視聴」（ 稲田, 2022）と呼ばれる映像作品を早送りで特定のシーンだけ注目して鑑賞する実践は、作品世界の全体性に注目せず瞬間瞬間のアトラクターによって刺激される快楽の充足を優先した鑑賞あるいは消費のあり方である。これは、東が言うところの、物語の全体性から乖離して個別的な「萌え」要素に反応するだけで、「問主体的な（引用者注 : 複雑な欲望の）構造が消え、各人がそれぞれ欠乏-満足の回路」（ 東, 2001, 127）に閉塞した状態である「動物化」が現実化していると言うこともできるだろう。

とはいえ、ラマールと東の議論が全く同一ということではない。映画理論における次元性の問題を吸収したラマールの議論は、主体が自身の目的や欲望に従ってさまざまな種類のイメージを操作するという現象の分析へと拡張可能だろう。レフ・マノヴィッチは『ニューメディアの言語の中で』、実写映画を実写の素材を特権的に使用するアニメーションの一形式と捉え、3次元性に重きを置く映画史観を反転させた ( Manovich, 2001, 302)。マノヴィッチはグラフィカル・ユーザー・インターフェイスを搭載したコンピュータ上でさまざまな種類のデジタルデータが統合的に操作可能であることをもってこの新しい美学的状況を議論の俎上に載せている。だが、本稿が見てきた通り、アトムのシールや 2.5次元舞台のように平面的なイメージがトランスメディア的に拡散している状況や、日本全国のアニメやマンガのキャラクターの像やキャラクターやアイドルが印刷されたアクリルスタンドを使ってセルフィーを撮る人々の存在を考えると、次元を跨いだイメージの操作という文化的実践はキャラクター文化の成立と同時に出現したと言える。だとすると、このとき主体はアニメや漫画のキャラクターという二次元のイメージと、自分たち自身やそれらが置かれた風景といった三次元のイメージとで、「分配的領域」を認知的に構成し、何らかの感情・情動的報酬を得ていると考えることはできる。このプロセス全体を東のシナリオに沿って欠乏-満足の回路を満たす行為だと考えることもできるかもしれないが、イメージを主体的に操作し合成している点で、ラマールの議論を援用してキャラクター文化を分析する道筋には主体の積極的な関与を見出す可能性が残されている。

## 平面と遊ぶ主体

主体がイメージに刺激されて欲望充足を行うだけの存在なのか、あるいはそれ以上の能動性を発揮しているのかという論点は、本稿の主体とイメージはどのように関わるのかという論点を一歩先に進めたものであるし、ここまで議論を参照してきた大塚も東も何らかの形で触れている論点である。大塚の物語消費と東のデータベース消費を対比し、なぜ「二次創作」という生産活動が「消費」と呼ばれてきたのかを論じた永田大輔の議論がこの点を理解する助けとなる（ 永田, 2022, 321-337）。大塚は、同じ「世界」をそれぞれの「趣向」として表出しているだけであるという意味で、小さな物語を作り出す二次創作は「生産」ではなく「消費」であるとしている（ 永田, 2022, 330）。したがって、永田が言うように「大塚が「物語消費論」をマーケティングに過ぎないというのは、彼が行う自己卑下などではなく、文字通りの意味でそうした概念なのである」（ 永田, 2022, 331）。永田は消費社会の中でオーディエンスの二次創作的生産行為が結局は物語全体を管理する製作者の掌の上で踊らされているだけであり、本当の意味では生産ではないという大塚の問題意識に光を当て直した。永田が指摘している通り、大塚が漫画の描き方講座などを展開し教育に注力しているのは、二次創作という消費にとどまるのではなく自分自身の作品を作り出せることを市民社会にとって重要なことだと考えているからだ（ 永田, 2022, 334）。

このような大塚の問題意識は、英語圏におけるメディア化した資本主義社会の中で市民の主体的な政治参加をめぐる問いと響き合うものである。カルチュラル・スタディーズやファン研究においては多くの論者が参加すなわち主体の能動性を強調する議論をしている。例えば、池田太臣によれば、アクセル・ブランスは Web 2.0の環境下で、コンテンツの生産者と消費者の区別が曖昧となった状況を「プロデュセイジ(produsage)」と呼んでいる。プロデュセイジは「さらなる改良を追求する、共同的で持続的な、既存のコンテンツの構築と拡張」と定義され、「プロデュセイジの環境においては、ユーザーが作り上げていくコンテンツ生産の過程は、生産者/利用者という区別が重要性を持たず、コンテンツを共有する利用者は、同時に生産者でもある」（ 池田, 2013, 107-119）とされている。同様の発想は岩渕功一の「プロジューマー(prosumers, producer-consumers)」と「アプロリーダー (approreaders, appropriator-readers)」にも見られる( Iwabuchi, 2010, 87-96)。カルチュラル・スタディーズのオーディエンスの能動性を評価する傾向が、ウェブ 2.0の技術的環境の中で浮上したものであり、したがってヘンリー・ジェンキンズの「参加型文化」( Jenkins, 1992; ジェンキンズ, 2021)とも轍を一にしている。

もちろん、ユートピア的にあらゆる種類の二次創作を市民的参加や能動性の発露とみなし肯定的に評価することはできない。しかし、ファン研究は一般的に、商品の消費を後々のより主体的な参加のための第一歩目として重視する傾向がある。例えば、英語圏におけるスラッシュ・フィクションや日本におけるやおいの二次創作は、少なくとも 1970年代から 1980年代の状況においては、男性向けに作られるコンテンツを改変することで女性たちが自分達の欲望を表現する道筋を作った ( Jenkins, 1992, 205)。こういった 20 世紀後半においては解放の可能性を読み込むことができた二次創作が、21 世紀現在においては原作のコンテンツ提供者によって促進される消費の一形態となっていることは否定できない（ ジェンキンズ, 2021, 155-169; 大塚, 2018）。「参加者の創造的能力に応じて段階的に用意され」（ 大塚, 2021, 136）た「参加」の手段を通して、ファンの能動的で主体的な参加がプラットフォーム提供者によってフリー・レイバー ( Terranova, 2000)として搾取されてしまう現実は存在する。

しかし、後期資本主義社会に生きている以上、このようなプラットフォームによるユーザーの搾取を完全に排除することは不可能であり、むしろ考えるべきは、資本主義の商品に囲まれている中からどのように市民的主体性を構想するかにある。資本主義的なコンテンツに刺激されたところからサークルや同人活動が立ち上がり、彼らの二次創作活動が後の作家を養成する母体となったことは事実である。そういった特殊な事例も重要だが、ほとんど多くの人々は作家や芸術家になるわけではないことを考えると、多くの人々が生活の中に浸透してくるキャラクターの魅力とその背後にある資本主義に対してどのように小さな抵抗を行うことが可能かを考察する必要がある。個々の人間がどのようにキャラクターという平面と戯れる中から市民的主体性を獲得するかを検討するためにも、

永田が言うように、「まんが・アニメ文化をめぐる具体的でローカルな実践記述が必要」（ 永田, 2022, 335）なのである。

その一つの事例としてコスプレを考えてみよう。コスプレとは、フィクション作品の登場人物の衣装や髪型を模倣し、登場人物になりきる文化実践である（ 貝沢, 2018, 57-73）。コスプレ実践と主体の関わりについて田中東子は次のように述べている。

自分自身を別のキャラクターに扮装させることを通じて行使されるこの顕示のための手段は、まったく風変わりな自己の提示方法でもある。自分自身の身体を使いながら、けれども自分そのものを他者に印象づけるのではなく、「キャラクター」という共通の記号を身にまとうことで「私」という自己を打ち消して約分してしまうのだから。その意味でコスプレは匿名化の装置でもある。「オリジナルな自己」を打ち消しつつ目立つ格好をできるという両義牲があるのだ。[…] 着脱可能な「レイヤー化」するアイデンティティの獲得。（ 田中, 2009, 36-37）

コスプレは平面的なイメージとしてのキャラクターをなんらかの形で利用し「私」という主体を立ち上げる複雑な文化実践である。キャラクターのイメージを身にまとい「着脱可能な「レイヤー化」するアイデンティティ」が獲得されるとき、マルヴィ的なシナリオとは異なる複雑な自己とイメージの関係が展開される。異性愛規範を強化する男性のまなざしと異なって、田中はコスプレイヤーたちの中に女性が多く、彼女たちが男性キャラクターを演じることが支配的な規範を撹乱する可能性を指摘している（ 田中, 2009, 39-41）。田中は、消費をするときにのみ市民として認めることを「ショッピングモール公共圏」として批判した議論をひいて、女性たちによるコスプレを新しい生きられた DIY文化として捉え直そうとしている。もちろん、このコスプレ自体も今では大衆的な認知を得て商業化しており、DIY性が後退し、結果として「ショッピングモール公共圏」に回収されてしまっている部分は否定できない。

だが、キャラクターという他者の平面的イメージを纏うコスプレが自己も複層化させながら、同時に現実の生活で対面する困難と折り合いをつけ対抗する方法として日本国外で実践されている事実もまた否定できない。例えば、東南アジアのイスラム教国家であるマレーシアとインドネシアでは、ヒジャブを漫画やアニメのキャラクターの髪色に合わせるヒジャブ・コスプレが実践されている。そうすることで、ムスリム女性たちがイスラム教の規範の中に留まりつつ自己表現をしている可能性が指摘されている ( Yamato, 2020)。また、北米ではコスプレをする際には必然的にエスニシティーの問題が可視化されることになり、「レースベンディング (racebending)」と呼ばれる文化実践が見られる ( Kirkpartick, 2019)。レースベンディングとはもともとメディアコンテンツの制作者が役の人種を変更することを批判する用語で、ハリウッドで非白人の役に白人をあてることを意味する「ホワイトウォッシュ」とほぼ同義で使われていた。しかし、#blackwash というハッシュタグと共にアニメや漫画のキャラクターを黒人として描き直すという文化実践も出現し、レースベンディングは主流のメディアにおける人種表象をマイノリティーが自己の生活にひきつける改変をも意味するようになった。小柄なアジア人であるブルース・リーが映画の中で大柄な白人男性を倒すことが 1970年代のアメリカ都市部のエスニック・マイノリティーの若者を勇気づけたように、漫画やアニメのキャラクターのイメージが現代の若者のアイデンティティ政治に取り入れられている ( Ongiri, 2002)。ヒジャブ・コスプレやレースベンディングといった文化実践の中では、キャラクターのイメージが素朴に同一化や欲望の対象とされるのではなく、操作・改変可能な平面的な図像として捉えられ、それを身に纏い遊ぶことで複雑な主体を作り出し、また社会との関係を変化させているのである。

## 結論

本稿はまず日本におけるキャラクター文化がその記号的な図像としての平面性をもっており、結果的にトランスメディア的に社会の中に拡散し浸透してきたことを指摘した。この平面性に対して人間主体がどのような関係を持っているのか、また平面性の向こう側に現実の世界がどのように立ち現れるのかを、日本における大塚英志や東浩紀の議論と英語圏の映画理論を参照して概観してきた。永田大輔による二次創作の中に生産と消費の要素が混在していることを指摘した整理を通過することで、主体と平面的イメージとの間に同様の能動性と受動性の混在した複雑な関係性が出現することを指摘した。キャラクターのイメージを纏うコスプレという文化実践は、それ自体が商業化され DIY性が後退していく点は否定できないものの、まさにそのようなイメージの利用を通して社会と交渉し主体を形成する試みである。これはコスプレという文化実践が特権的に重要という意味ではなく、文化実践が常に商業化されていくという資本主義社会の条件の中で、例えばアクリルスタンドを使ったイメージの生産といった比較的新しい文化実践を、人々がどのように商業的に提供されるイメージと折り合いをつけながら自身の生を豊かにするための手段として利用しているのかを注視し続けることが必要なのである。

## データ可用性

本論文には関連データがない。
