# Supplementary material for: Subject playing with flat images: Transmedia spreadability of <i>anime</i> and <i>manga</i> character images
Source: F1000Res. 2023 Oct 13;12:191. [Article in Japanese] Originally published 2023 Feb 20. [Version 2] doi: 10.12688/f1000research.129643.2 (PMC10905127; doi:10.12688/f1000research.129643.2)
Supplement: Peer review history

# Reviewer report: Reviewer response for version 2

**DOI:** 10.5256/f1000research.156350.r214874

**Published:** 2024-02-16

**Authors:** Daisuke Nagata

**Affiliations:** 1 Faculty of Humanities, Meisei University, Tokyo, Japan

今回の改訂で大きく可読性も上がっており、「承認」で問題ないと考えます。複数の領域を接続させていくうえでの視座やキャラクター論やポピュラーカルチャーを評論していくうえでの指針（イメージだけを論じるのではなく、主体とイメージの関係に視点を移すこと）としても今後参照されていく論文であると考えます。3点ほど細かな指摘をしますが、対応いただいても対応いただかなくても「承認」といたします。

**(1)** **方法の項目について**

 分野によるのかもしれませんが、この項目に書かれているのは全体の構成といったものに近く、方法という言葉には若干の違和感を感じました。やられているのは通常の論文でいう2節では〜について論じる。3節では〜について論じるといった記述に該当するものだとは思うので、ワーディングをできれば再考いただきたいです。

**(2)** **伊藤の議論の「あいまいさ」が何であるか不明瞭に見える**

 伊藤から岩下へ議論がうつる際に伊藤の議論に曖昧さが残るとあるがどのようにあいまいなのかが読者には不明なように思う。どのように曖昧だったから岩下のような議論がされたのかについて一文ほど加筆いただきたい。

**(3)** **マルヴィ・ラマールの説明前後の記述**

 縫合理論も難解なので可能であればもう少し記述を柔らかくしてもらいたいが、難しいかとも思うので不明語を減らしてほしい。例えばマルヴィが出てくる直前に「想像界的」な快楽とあるがこれが何なのかは読み進めても確定しきれないので説明が必要なように思う。またアトラクターとコーぺーレーターの説明もこれが萌えとオタクのような関係に接続するということはまだわかるのだが、アトラクター・コーペーレーターがそもそも何なのか（一般的な言葉でいうと何なのか、どのような物理用語なのか）もわかりにくい（特にコーペレーター）。可能であれば加筆いただきたい。

（該当する場合は要回答）統計分析および解釈は適切ですか。

対象外（統計を使っていない）

本研究は明確かつ正確に提示されたものであり、最新の文献を引用していますか。

はい

方法と分析について第三者による再現が可能となるよう十分な詳細が提示されていますか。

一部該当

結果の基礎となるソースデータはすべて入手可能で再現性を十全に保証していますか。

ソースデータは不要

結論は結果により妥当な裏付けを得ていますか。

一部該当

研究設計は適切で学術的価値がありますか。

一部該当

Reviewer Expertise:

文化社会学・メディア論・アニメーション文化論

I confirm that I have read this submission and believe that I have an appropriate level of expertise to confirm that it is of an acceptable scientific standard.

# Reviewer report: Reviewer response for version 1

**DOI:** 10.5256/f1000research.142340.r189344

**Published:** 2023-08-18

**Authors:** Hiroshi Morishita

**Affiliations:** 1 Faculty of Letters, Soka University, Hachioji, Tokyo, Japan

総論

 　本論文は、近年の日本で書かれてきたマンガ・アニメのキャラクター論を概観し、その上でそれを、特に欧米における映画研究やファン研究など、関連する他分野にて培われてきた議論と接続することを試みたものである。この作業を通じては、「キャラクター・イメージが力を持って人を動員するメカニズム自体」（p.3）を議論の俎上に載せることが意図されている。最終的には、トーマス・ラマール『アニメ・マシーン』（2009年）を経由しながら、欧米系の映画理論の主張とは異なる、「平面的なイメージとしてのキャラクターと鑑賞する主体との間に発生する複雑な関係性」（p.3）を明らかにし、受け手の実践が持ち得る可能性に注意を促している。

 　論旨は明快である上、さまざまな研究領域を横断・整理している本論文は、今後のキャラクター研究の発展にも資するものである。その意味で十分に価値があるものであることは間違いないが、種々の研究を接続する形で議論が紡がれているため、独自の知見に乏しいように感じられる部分があることは否めない（これについては、すでに永田も、前半部分について「東の議論のパラフレーズにとどまっているところが多いようにも一見すると見えてしまう」（p.13）と指摘している）。先行研究の著者の想定とは異なる形でその議論が持ち得る可能性を探っていることをわかりやすく示しておけば、こうした点は多少なりとも軽減されるのではないか。

 　「大塚と東はともに作品というものが存在することを前提としてその上で鑑賞者とその作品世界との関わりを論じているが、本稿が注目するのは作品世界の境界線を超えて我々の日常生活の中にキャラクターが氾濫しているという事実である」（p.6）と、筆者は述べている。この比較は正確だが、彼らの著作そのものの方向性が筆者の問題関心とは異なることには、もう少し言葉を費やしてもよいだろう。大塚はもちろん、東の『ゲーム的リアリズムの誕生』（2007年）も、小説が読まれる環境それ自体が持つ効果を重視する「環境分析」的な読解によってさまざまなキャラクター小説やゲームを論じるものである。つまり、キャラクターそれ自体をフラットに分析するものではなく、本質的には詩学ないし作品論というべきものだろう。筆者が、作品批評や文化批評、作品論などとはちがう形でのキャラクター論の構築を図っているということと、その積極的な意義とは、この節（「平面の向こう側」）において今以上に強調されてよい。

 「キャラクター論における平面性」に関して

 　その上で、以下では個別の議論に触れておきたい。

 　「キャラクター論における平面性」の節では、「キャラクターが比較的簡単な線画として平面的に図像化されていることが、そのトランスメディア的拡散を可能にしている」（p.5）と結論づけられているが、もう少し慎重な記述が必要ではないだろうか。これに関しては、永田も挙げていたが、やはり足立加勇「人間の表象としてのキャラクターとファンのコンテクストとしてのキャラクター――消費者集団の社会活動が生み出すキャラクターの二面性」（永田大輔、松永伸太朗編『アニメの社会学　アニメファンとアニメ制作者たちの文化産業論』ナカニシヤ出版、2020年）が重要だろう。

 　この論考で、足立は、初音ミクなどの例を挙げ、伊藤のいう「キャラ」が、特に消費者の行為によって形づくられたコンテクストによって創出されるものであることを示している。集団内での合意が重要である以上、論理的には、「比較的簡単な線画」ではない形で造形されているキャラクターであっても、トランスメディア的に拡散することは十分に可能である。例えば、アニメ『鉄腕アトム』（1963-66年）と並ぶ1960年代の日本を代表するキャラクターコンテンツとして、『ウルトラマン』（1966-67年）等、怪獣や巨大ヒーローが登場するものがある。実写作品である以上、怪獣の体表などのディテールは本来かなり細かいものではあるが、それらもまたソフトヴィニール製の人形などの形で、スタインバーグの挙げるアトムのシールの場合と同様に、子どもたちの生活に浸透していった。つまり、キャラクターが「比較的簡単な線画」で造形されていることは、送り手や受け手のあいだでの合意形成を容易にするものではあるが、それ自体が「トランスメディア的拡散を可能に」する必須の条件ではない。そのような可能性も考慮に入れる必要があるだろう。

 「平面と遊ぶ主体」に関して

 　総論の項で述べたとおり、本論文は終盤にて、キャラクターを用いた受け手の実践に、「複雑な主体を作り出し、また社会との関係を変化させ」（p.11）る可能性を見出している。先行研究の読み替えと精緻化から一歩進んで、論者自身の価値判断が展開されているといえよう。

 　このように自らの見解を提示するにあたり、筆者は、二次創作を「生産」ではなく「消費」であると見なす大塚の見解を取り上げつつ、「実際に二次創作活動が後の作家を養成する母体となったことも否定できない」（p.11）と、これを退けている。筆者のスタンスははっきりしているが、このように論じるのであれば、現在のいわゆる「オタク文化」におけるプラットフォームを通じた動員に関する議論にも踏みこむべきではないか。現在の大塚は、必ずしも学術的な論考に限らない形で、しばしばこうした問題を検討しているからである。

 　大塚は、角川歴彦のもとでプラットフォームに応じて最適化したヴァリアントを生成する形でのメディアミックス展開が成立したと位置づけている。現在のKADOKAWAも、「ユーザーのコンテンツ制作を誘発し回収するシステム」としてこの延長上にある、というのが彼の分析である。その上で彼は、物語を生成するシステムそのものがだれかに管理される事態そのものに警鐘を鳴らす（『メディアミックス化する日本』イースト・プレス、2014年、p.35）。現在のマンガやアニメ、ゲームなどでは、「ファン活動そのもの」が「エコサイクルとして組み込」まれることが前提となっており、同人誌活動を伴わない場合でも、「アニメーションの舞台の聖地巡礼など、参加者の創造的能力に応じて段階的に用意され、拡張している点で特徴的である」（『シン・モノガタリ・ショウヒ・ロン　歴史・陰謀・労働・疎外』星海社、2021年、p.136）。

 　こうした大塚的な立場に立つならば、二次創作活動がのちの作家の母体となっていたとしても、プラットフォームによる動員と搾取という問題は揺るがないことになる。また筆者は、田中東子「コスプレという文化」（2009年）を引きながら、「「私」という主体を立ち上げる複雑な文化実践」（p.11）としてコスプレを捉え直しているが、おそらく2009年当時と現在とでもすでに状況は異なっているだろう。コスプレという文化が広がるにつれてDIY性が後退し、「参加者の創造的能力に応じて段階的に用意され」た「参加」の手段のひとつとなり、「ショッピングモール公共圏」に回収されている部分もあるはずである。

 　いうまでもなく大塚の立論に従う必要はないが、その主張に触れているにもかかわらず、プラットフォームの問題に立ち入らないのはいささかフェアではないように思われる。キャラクター論を精緻化する作業を通じて、東の「動物化」という指摘や大塚の倫理的な批判を乗り越える形で受け手の文化実践の意義を浮かび上がらせることができれば、本論文の意義もより鮮明になるだろう。

 誤字・脱字

 　以下は単純なミスの指摘である。確認・修正されたい。

 p.3　「方法」の節の上から5行目：「伊東」→「伊藤」

 p.6　上から3行目：「強調しすることで」→「強調することで」

 p.7　下から7行目：「映画的リアリズムという規範からは三次元的間が重視され」→「映画的リアリズムという規範からは三次元的空間が重視され」？

 ・足立加勇「人間の表象としてのキャラクターとファンのコンテクストとしてのキャラクター――消費者集団の社会活動が生み出すキャラクターの二面性」永田大輔、松永伸太朗編『アニメの社会学　アニメファンとアニメ制作者たちの文化産業論』ナカニシヤ出版、2020年、pp.204-220

 ・大塚英志『メディアミックス化する日本』イースト・プレス、2014年

 ・大塚英志『シン・モノガタリ・ショウヒ・ロン　歴史・陰謀・労働・疎外』星海社、2021年

（該当する場合は要回答）統計分析および解釈は適切ですか。

対象外（統計を使っていない）

本研究は明確かつ正確に提示されたものであり、最新の文献を引用していますか。

はい

方法と分析について第三者による再現が可能となるよう十分な詳細が提示されていますか。

はい

結果の基礎となるソースデータはすべて入手可能で再現性を十全に保証していますか。

ソースデータは不要

結論は結果により妥当な裏付けを得ていますか。

はい

研究設計は適切で学術的価値がありますか。

はい

Reviewer Expertise:

ポピュラー･カルチャー研究

I confirm that I have read this submission and believe that I have an appropriate level of expertise to confirm that it is of an acceptable scientific standard.

# Response

**Published:** 2023-09-21

**Authors:** Kohki Watabe

**Affiliations:** University of Tsukuba, Japan

森下達様

 今回は査読をお引き受けいただき誠にありがとうございます。論文の細部まで読んでいただき、私が十分に議論しきれなかった部分もフォローするコメントをいただき大変感謝しております。以下、ご指摘の内容への応答を簡単にコメントいたします。

 【大塚・東の作品論との対比について】

 ・ご指摘のとおり本稿はキャラクターのトランスメディア的拡散が焦点で、大塚・東の作品論的関心の持ち方とは立ち位置が異なるものです。

 ・ご教示いただいた足立加勇氏の論考はこの立場の違いを明示するのに大変参考になる論考でしたので、本稿で参照いたしました。

 【「比較的簡単な線画」でなくてもトランスメディア的拡散が起こることについて】

 ・この点はご指摘のとおりで、「比較的簡単な線画」はトランスメディア的拡散が起こるための「十分条件」ではありません。

 ・「平面的キャラクターのトランスメディア的拡散」の節の最後に一段落加筆し、この点について留保をつけました。

 【新しい文化的実践が資本主義的プラットフォームに組み込まれることについて】

 ・この論点は初稿の段階でうまく言語化できていなかったのですが、森下様のご指摘によって、論点が明確になりました。

 ・ご指摘の通り（1）プラットフォームの力は無視できないこと、（2）新しい文化的実践もプラットフォームに組み込まれてDIY性が後退すること、は現実に起きていることであり、これらを過小評価することはできないと考えています。

 ・その上で、これらの論点を加え、「平面と遊ぶ主体」の節を大幅に改稿いたしました。

# Reviewer report: Reviewer response for version 1

**DOI:** 10.5256/f1000research.142340.r163899

**Published:** 2023-03-24

**Authors:** Daisuke Nagata

**Affiliations:** 1 Faculty of Humanities, Meisei University, Tokyo, Japan

**総論**

 本論は日本発のマンガ・アニメのキャラクター・イメージの拡散を素材に(1)日本で練り上げられてきた議論を整理したうえで、欧米のフィルムスタディーズの議論とトマス・ラマールの議論を接続させつつ論じ、そこで「主体」と平面的な対象の関係という議論の視点を提示したこと、（2）大塚らの議論を基礎としてカルチュラル・スタディーズで問題となってきたアクティブ・オーディエンスをめぐる議論の文脈でいかなる可能性がキャラクター論にあるのかをコスプレなどの具体的な文献をめぐる研究を媒介とさせつつ論じている。

 そうした点で非常に野心的な論考であるといえるが、系譜の異なった複数の議論を接続させていることから、(1)と(2)の内部と(1)と（2）の接続のそれぞれについて方法・結論・研究設計に関わる形でいくつか明確化させた方がよいように思われる。

**フィルムスタディーズと東らの議論の接続に関して**

 (1)のパートで最も気になった点として対象と主体の関係こそが重要だという結論が前半で出されており、そこから映画研究の知見を接続させているが、基本的には東の議論のパラフレーズにとどまっているところが多いようにも一見すると見えてしまうことである。その接続自体に一定の意義があることはわかるのだが、その接続を経たことによって、キャラクター文化が拡散し我々の社会に根差すという事態に対して東の議論の知見がどのように変わりうるのかという点についてもう少し明確にしうるのではないだろうか。

 読んでいる段階で東の議論の整理がある程度明確であるからこそ、ある程度答えが、出ているように思えてしまい、接続させること意義を途中で見落としてしまう印象がある。

 この解決方法としては二つの方向性があるように思われる。それは東の議論の限界や不明確に見える点をもう少し明示化して答えるという方向性である。もう一つの方向性は先に（須川等の議論を紹介したあとに）映画を事例にした主体と対象の関係の議論を紹介し、そのうえで東の議論を解釈しどこまでが映画と主体の解釈の問題として論じられ、どこで息詰まるのかを論じたうえでラマールにつなげるという方向性も存在するように思う。それぞれご検討いただきたい。

**本筋の議論に必要な議論の絞り込みに関して**

 もう一つ、本論の議論の核は主体と対象世界の関係論であるはずなのでその意義をより明確にさせるパートを中心にした方がよいように思う。そのために例えば伊藤剛のキャラ論はこの分野の重要研究ではあるが、本論のキャラに人々はどのようにかかわるのかという問いの視点から考えたときには迂遠な説明である印象が否めないため、もう少し縮減した説明することで意義が明確となりやすいと思われる。また、必須ではないが伊藤のキャラクター論と主体の関係を論じるうえでは足立加勇 2020「人間の表象としてのキャラクターとファンのコンテクストとしてのキャラクター――消費者集団の社会活動が生み出すキャラクターの二面性」(永田大輔・松永伸太朗（編）『アニメの社会学――アニメファンとアニメ制作者たちの文化産業論』ナカニシヤ出版）が参考になると思われる。

 また原形質の議論も重要であるが本筋とは外れるため必要があるのか疑わしい説明であるように思う。ここの流れで重要なのは本論の問いが「人々がキャラクターイメージが力を持って人を動員するメカニズム自体の検討」である以上、キャラクター文化が偏在すると同時に我々がそれを同じキャラクターとして享受可能であり、萌えて楽しむことができるのはなぜかという問いを投げかけて、キャラクターの問題を（平面の）対象と人間主体との関係として捉え返す必要があることが提示されることにあるはずだからである。

**情動論とアクティブオーディエンスの関係について**

 後半部分のラマールの整理から「平面と遊ぶ主体」までの接続（要するに(1)と(2)の接続）の問題についても少し唐突な印象を感じた。動物に過ぎないのかと主体の能動性の問題の記述が唐突に感じるし、この問い自体は例えば東の動物化などでダイレクトに接続されているものではない（のちにつなげようとした議論が存在したのは事実だと思うが）。一案として動物化論と英語圏のアクティブオーディエンスとの接続をするために「平面と遊ぶ主体」の例えば～からの部分とこういった～の段落の部分を入れ替えるなどすると両者の接続がさせやすいのではないだろうか。

 本論の問いはA（良きにしろ、悪しきにしろ）「人々がキャラクターイメージが力を持って人を動員するメカニズム自体の検討」とB「キャラクターの拡散」の双方を射程に入れたものである。だが、最後の節では「悪しきにしろ」ではない形でいかなる形で対象世界と主体の関係は可能なのかという点に踏み込んでいるように見える。それ自体は論者の立場としては重要だと思うが、その立場に踏み込むことが明示されないまま議論が進んでいる印象があるので議論の移行点が明示される必要があるのではないだろうか。

 ・足立加勇 2020「人間の表象としてのキャラクターとファンのコンテクストとしてのキャラクター――消費者集団の社会活動が生み出すキャラクターの二面性」(永田大輔・松永伸太朗（編）『アニメの社会学――アニメファンとアニメ制作者たちの文化産業論』ナカニシヤ出版

（該当する場合は要回答）統計分析および解釈は適切ですか。

対象外（統計を使っていない）

本研究は明確かつ正確に提示されたものであり、最新の文献を引用していますか。

はい

方法と分析について第三者による再現が可能となるよう十分な詳細が提示されていますか。

一部該当

結果の基礎となるソースデータはすべて入手可能で再現性を十全に保証していますか。

ソースデータは不要

結論は結果により妥当な裏付けを得ていますか。

一部該当

研究設計は適切で学術的価値がありますか。

一部該当

Reviewer Expertise:

文化社会学、映像文化論、社会学、メディア論、アニメーション研究

I confirm that I have read this submission and believe that I have an appropriate level of expertise to confirm that it is of an acceptable scientific standard, however I have significant reservations, as outlined above.

# Response

**Published:** 2023-09-21

**Authors:** Kohki Watabe

**Affiliations:** University of Tsukuba, Japan

永田大輔様

 査読の労をとっていただきお礼申し上げます。丁寧かつ建設的な査読コメントで大変参考になりました。以下、ご指摘いただいた主要な点についてどのように改稿したか、簡単に応答いたします。

 【議論の絞り込みについて】

 ・「原形質性」についてはおっしゃるとおり議論の本筋からは外れるので、大幅にカットしました。

 ・伊藤剛のキャラクター論については、確かに議論全体の流れからすると確かに迂遠だとおもいましたので、彼のキャラクター論全体ではなく本稿の焦点である「キャラ」の部分に絞り込んだ引用をするように整理しました。

 ・ご教示いただいた足立加勇氏の論考はおっしゃる通り重要な論点を提示しているものですので、本稿に引用しました。

 【フィルムスタディーズと東らの議論の接続に関して】

 ・初稿の段階では東の議論とラマールの議論の差異が明示されていないというのは、自分では気づかなかった点で、指摘されて大変納得しました。この点ご指摘いただいて大変ありがたいです。

 ・ご提案いただいた改稿方針2つのうちの前者の方針に従い、「分配的領域を見る脱一点透視図法的主体」と題する節の終わりに長めの段落を追加し、東とラマールを対比して整理しました。

 【情動論とアクティブオーディエンスの関係について】

 ・ご指摘の中で私の議論を整理していただき、その内容によって私の理解も深まりました。ありがとうございます。

 ・前述の東の議論を整理する中で、最後の「平面と遊ぶ主体」の節との接続もクリアになり、ご提案いただいた段落の入れ替え等の修正と合わせて、大幅に内容を改稿しました。
